# Depressive Symptoms Among Children and Adolescents in China During the Coronavirus Disease-19 Epidemic: A Systematic Review and Meta-Analysis

**DOI:** 10.3389/fpsyt.2022.870346

**Published:** 2022-04-08

**Authors:** Jianghe Chen, Kun Yang, Yujia Cao, Yun Du, Ningqun Wang, Miao Qu

**Affiliations:** ^1^Department of Neurology, Xuan Wu Hospital of Capital Medical University, Beijing, China; ^2^Department of Neurology, Third Affiliated Hospital, Beijing University of Chinese Medicine, Beijing, China; ^3^Evidence-Based Medicine Center, Xuan Wu Hospital of Capital Medical University, Beijing, China; ^4^Xian Fifth Hospital, Xi’an, China; ^5^Department of Rheumatology Immunology, Dongfang Hospital, Beijing University of Chinese Medicine, Beijing, China

**Keywords:** COVID-19, children and adolescent, China, depression, meta-analysis

## Abstract

**Background:**

The Coronavirus Disease-19 (COVID-19) pandemic negatively impacts mental health. Some published studies have investigated the prevalence of depression among children and adolescents in China during the pandemic. However, the results vary widely. We aimed to systematically analyze and estimate the prevalence of depressive symptoms and attempted to reveal the reasons for prevalence variety in previous studies.

**Methods:**

Published studies were searched in PubMed, Embase, Cochrane Central, the Chinese Scientific Journal Database (VIP Database), China National Knowledge database (CNKI), and the WanFang database from December 2019 to May 2021. The quality of all included studies was assessed by the Joanna Briggs Institute (JBI) checklist and the American Agency for Health Care Quality and Research’s (AHRQ) cross-sectional study quality evaluation items. Meta-analysis was performed using random-effects modeling.

**Results:**

Of the 1,708 references screened, 13 related reports that involve 41,729 participants were included. The results suggested that the pooled prevalence of depressive symptoms among Chinese children and adolescents during the COVID-19 epidemic was 28.6%. Subgroup analyses showed that the pooled prevalence was highest among the studies using the Patient Health Questionnaire (PHQ)-9 (46.8%) and lowest among these using Depression Self-Rating Scale for Children (DSRSC) (11.4%). All studies using PHQ-9 set the cutoff at 5 points instead of 10. The pooled prevalence of studies that include primary school students was lower (16.5%) than that of studies excluding primary school students (39.1%).

**Conclusion:**

The meta-analysis suggests that depressive symptoms were relatively prevalent among Chinese children and adolescents during COVID-19, especially among the secondary school students. The suitable screening tools and cutoff should be carefully chosen in the survey.

## Introduction

Depression severely impacts the mental health of children and adolescents. According to previous studies, depression in children and adolescents may have devastating consequences (1–3).

Early evidence highlighted that depression symptoms are very common among children and adolescents in China, the prevalence was estimated to be 22.2% (4), before the outbreak of Coronavirus Disease-2019 (COVID-19). Since the first case of COVID-19 was reported in Wuhan, Hubei, China (5), it had spread rapidly among provinces in China in a short time. At the same time, the COVID-19 was also reported around the world (6). The Chinese government implemented social distancing, home confinement, and school closure measures to control the spread of the infection. All Chinese children and adolescents were separated from society and schools from January to April 2020 (7). The prolonged home confinement is associated with a range of negative outcomes for children and adolescents, such as decreased social interactions with peers, reduced physical activity, increased conflicts with parents, and academic pressures due to sudden changes in traditional learning methods (8, 9). These might lead to a persistent impact on the mental health of children and adolescents and eventually contribute to the occurrence of depressive symptoms (10).

There have been some cross-sectional studies on the prevalence of depression among children and adolescents in China during the COVID-19 epidemic. However, the results vary widely, some studies found that it was as high as 52.4% (11), while others found that it was as low as 10.4% (12). Given the large disparity in previous cross-sectional studies since the beginning of the pandemic, the purpose of this review was to provide an estimate of the prevalence of depressive symptoms and to explore the reasons for prevalence variety in previous studies (e.g., differences in the application of screening scales). This is of great significance for further intervention and may also play a certain role in the design of the future studies on depression in children and adolescents who experience major public events.

## Methods

This study was conducted in accordance with the requirements and standards set forth in the Preferred Reporting Items for Systematic Reviews and Meta-Analyses (PRISMA) framework (13). All data analyses were based on the original study, so no additional ethical approvals and consent forms of the participants were required.

### Search Strategies

A systematic search was performed to identify published studies on the prevalence of depressive symptoms in Chinese children and adolescents during the COVID-19 outbreak. We searched 6 major digital databases. The English databases were PubMed, Embase, and Cochrane, and the Chinese databases were China National Knowledge Infrastructure (CNKI) database, WanFang database, and VIP database to find all of the articles that met the criteria. In the search strategy, medical keywords (Medical Subject Headings; MeSH) were used to find relevant research published at all times: “Depression*,” “Depressive Symptoms*,” “Depressive Disorder*,” “Mental Health*,” “COVID-19*,” “Adolescent*,” “Child*,” “Youth*,” “Teenager*,” “Student*,” “China*,” “Prevalence*,” “Ratio*.” Some entry terms with similar expressions are omitted here. Bibliographies and citations of the related articles and review studies were also screened for other potential articles.

### Inclusion and Exclusion Criteria

All of the original studies were included if they met the inclusion criteria listed below: (1) cross-sectional study of depression symptoms among children and adolescents under the age of 20 and over the age of 6 in mainland China; (2) the prevalence statistics of depression were calculated based on related articles; (3) using standardized self-assessment scales; (4) the study subjects were limited to children and adolescent; and (5) cross-sectional study was conducted during the COVID-19 pandemic.

The following publications were excluded: (1) published reviews, meta-analysis, case reports, comments, protocols, letters, and editorials; (2) the subjects were patients with different medical conditions or comorbidities, such as schizophrenia, circulatory disorder, or bipolar disorder; and (3) the participants of the study included people infected with the new coronary virus. Any disagreement at each stage was resolved through consensus.

### Data Extraction

Two reviewers (JC and YC) independently screened titles and/or abstracts, then searched the full text of studies and independently assessed them according to inclusion and exclusion criteria. Two reviewers independently collected critical data information using a standardized data extraction form. After one independent reviewer completed the review results, the other reviewer would conduct a thorough and rigorous review of the review results. If the two reviewers could not reach a consensus on the review results, then the third reviewer (YD) would conduct the final review and a consensus would be reached.

### Quality Assessment

Each of the original studies was assessed by the JBI checklist (14) and American Agency for Health Care Quality and Research’s (AHRQ) cross-sectional study quality evaluation items (15). JBI’s quality evaluation tool for prevalence research includes 9 items, which evaluate the overall quality of prevalence research in terms of sampling methods, research objects, data collection, and analysis methods. The item is scored 1 point if the answer “yes,” and scored 0 point if answer “no,” “not clear,” or “not applicable.”

The AHRQ’s cross-sectional study quality evaluation items were compiled by the AHRQ to assess the quality of cross-sectional research. It contains 11 items, the answer “yes” is scored 1 point, and “no” or “not clear” is scored 0 points. All included studies were classified as having “low” (0–3 points), “medium” (4–7 points), or “high” (8–11 points) methodological quality. Discrepancies in the scores of included studies were resolved through discussion to reach a consensus.

### Statistical Analyses

Stata version 16.0 (Stata Corp., College Station, TX, United States) was used for all statistical analyses. Prevalence estimates of depression were calculated by pooling the study-specific estimates using random-effects. The model could figure out an overall estimate weighted by sample size under the assumption of statistical heterogeneity between studies, which greatly reduced the impact of statistical heterogeneity (16). We chose random-effects because the expectations of the study effects were unlikely to be identical and the variability across the studies was expected.

### Assessment of Heterogeneity

The heterogeneity across the studies was assessed by determining the *I*^2^ statistic to quantitatively measure the inconsistency across studies (17). Exploratory subgroup and meta-regression analyses were conducted to examine the possible sources of heterogeneity, and sensitivity analyses were performed to assess the robustness and stability of the results.

### Assessment of Reporting Biases

Reporting biases were assessed by scrutinizing the protocols of the included studies (18). Potential publication bias was assessed by visually inspecting the funnel plots and quantified by Egger’s and Begg’s tests (19).

## Results

### Study Characteristics

**FIGURE 1:**
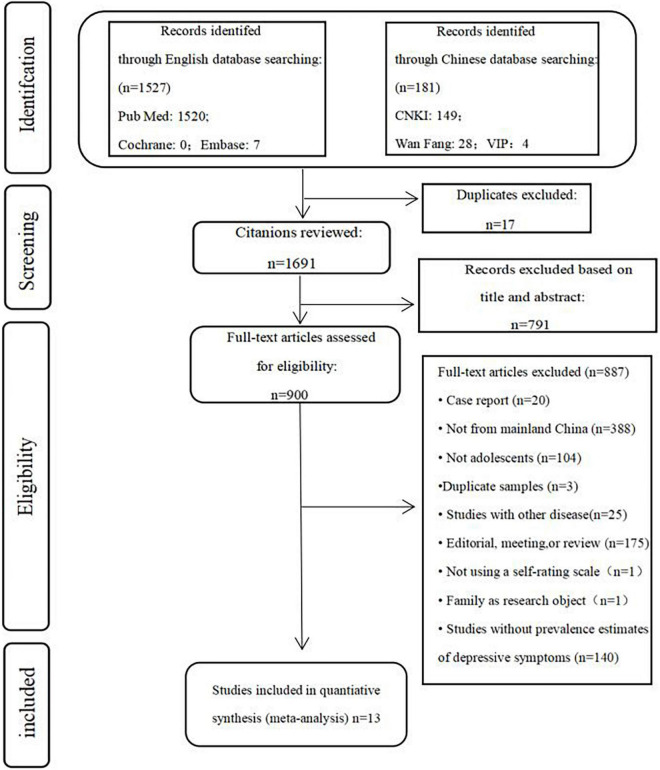
PRISMA flowchart of study selection.

We initially identified 1,708 articles by searching 6 academic databases, of which 1,691 were evaluated after removing duplicates. Of these, 900 articles were retained after the title and abstracts were screened. After reviewing the full text, 13 papers meeting the inclusion criteria were included in the analysis. Of the 887 articles excluded, 20 were case reports, 388 samples were not from China, 104 were not children and adolescents, 3 studies reported the same samples, 25 studies focused on other specific diseases, 175 were editorials or reviews, 1 did not use self-rating scale, 1 took the family as the research object, and 140 did not provide an assessment of the prevalence of depression. The flowchart of the selection process is shown in Figure 1. The characteristics of all included studies are summarized in Table 1. In these 13 studies (8, 11, 12, 20–29), a total of 41,729 children and adolescents had participated, with sample sizes ranging from 396 to 9,554. The publication year was between 2020 and 2021. The quality assessment shows that 6 articles were of high quality and 7 articles were of medium quality.

**TABLE 1:** Characteristics of the 13 studies of depressive symptoms in the meta-analysis.

References	The time of the investigation	Scale	Grades	Sample size	Case	Prevalence	AHRQ score	JBI score
Zhou et al. (20) and Chan et al. (52)	From March 8th, 2020 to March 15th, 2020	PHQ-9	J,S	8,079	3,533	43.7%	9	8
Zhang et al. (11) and Duan et al. (25)	From May 1st, 2020 to May 7th, 2020	PHQ-9	S	1,018	533	52.4%	7	7
Chen et al. (21) and Deeks et al. (56)	From February 22th, 2020 to March 8th, 2020	PHQ-9	J,S	7,772	3,334	42.9%	9	8
Yang et al. (22) and Duan et al. (25)	From May 1st, 2020 to May 5th, 2020	PHQ-9	S	838	418	49.9%	8	7
Cui (7) and Xie et al. (23)	From February 28th, 2020 to March 5th, 2020	CDI	P	1,784	403	22.6%	7	8
Tang and Hongwei (24) and Clayborne et al. (37)	About 2 months after the outbreak of COVID-19	CDI	P,J	873	102	11.7%	6	6
Duan et al. (25) and Clayborne et al. (37)	N.A.	CDI	P,J,S	3,613	805	22.3%	6	8
Chen et al. (26) and Chan et al. (52)	From February 20th, 2020 to February 27th, 2020	CESD	J,S	9,554	3,498	36.6%	10	9
Tang and Hongwei (24) and Deeks et al. (56)	From March 13th, 2020 to March 23th, 2020	DASS-21	P,J,S	4,342	857	19.7%	10	7
Zhang et al. (27) and Deeks et al. (56)	From April 7th, 2020 to April 24th, 2020	DASS-21	J,S	1,025	226	22.0%	7	6
Xiao-rong et al. (28) and Deng et al. (42)	From January 26th, 2020 to January 28th, 2020	DASS-21	S	1,399	366	26.2%	6	7
Chen et al. (21, 29)	From April 16th, 2020 to April 23th, 2020	DSRSC	P,J	1,036	122	11.8%	6	7
Yue et al. (12) and Chan et al. (52)	From February 6th, 2020 to February 8th, 2020	DSRSC	P,J,S	396	41	10.4%	8	6

*P, primary school; J, junior high school; S, senior high school; PHQ-9, the Patient Health Questionnaire; CDI, Children’s Depression Inventory; CES-D, Center for Epidemiological Studies Depression; DASS-21, Twenty-one-item Depression Anxiety Stress Scale; DSRSC, Depression Self-Rating Scale for Children; JBI, Joanna Briggs Institute’s critical appraisal checklist for studies reporting prevalence data.*

### Prevalence of Depressive Symptoms Among Children and Adolescents in Mainland China During the Coronavirus Disease-19 Epidemic

**FIGURE 2:**
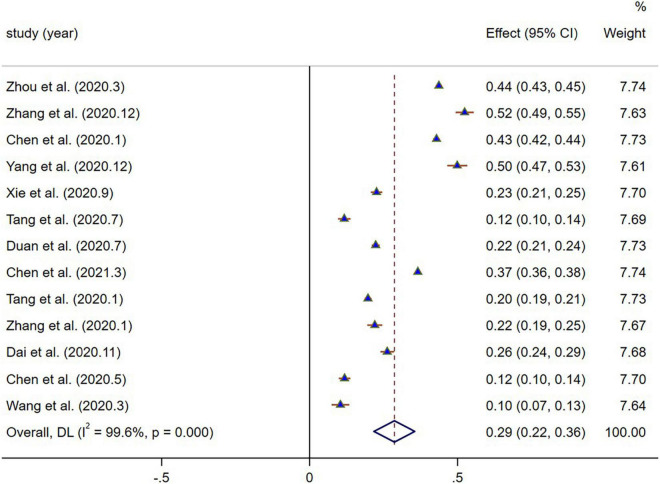
The forest plot of the prevalence of depressive symptoms.

The prevalence of depressive symptoms of all included studies was described, it ranged from 10.4 to 52.4% (Table 1). The overall pooled prevalence of depressive symptoms in children and adolescents was 28.6% (95% CI: 21.7–35.5%, *I*^2^ = 99.6%, *p* < 0.001), showing significant heterogeneity among studies (Figure 2).

### Heterogeneity Assessment and Subgroup Analysis

The heterogeneity across the studies was assessed by determining the *I*^2^ statistic to quantitatively measure the inconsistency. We stratified the results by screening tool, and grades are summarized in Table 2. Significant differences were found in the prevalence of depressive symptoms based on different scales. Four studies used the Patient Health Questionnaire (PHQ-9) scale, the pooled prevalence was estimated as 46.8% (95% CI, 43.6–50.1%; *I*^2^ = 93.3%). Three studies used the Children’s Depression Inventory (CDI) scale, it was estimated as 18.9% (95% CI, 12.5–25.3%; *I*^2^ = 97.4%). One study used the Center for Epidemiological Studies Depression (CESD) scale, which was estimated as 36.6% (95% CI, − 35.6 to 37.6%; *I*^2^ = 100%). Three studies used the Twenty-One-Item Depression Anxiety Stress Scale (DASS-21) scale, which was estimated as 22.6% (95% CI, 18.6–26.5%; *I*^2^ = 91.9%). Two studies used the Depression Self-Rating Scale for Children (DSRSC) scale, which was estimated as 11.4% (95% CI, 9.7–13.0%; *I*^2^ = 0.0%). The pooled prevalence of depressive symptoms was highest with the PHQ-9 (46.8%; *p* < 0.001) and lowest with the DSRSC (11.4%; *p* > 0.05).

**TABLE 2:** Subgroup analysis of the prevalence of depressive symptoms.

Subgroup	No. of	No. of	No. of	Pooled prevalence of	(*I*^2^)
	Studies	Participants	Cases	Depressive (95% CI)	
Overall	13	41,729	14,238	0.286(0.217∼0.355)	99.6%
**Scale**					
PHQ-9	4	17,707	7,818	0.468(0.436∼0.501)	93.3%
CDI	3	6,270	1,310	0.189(0.125∼0.253)	97.4%
CESD	1	9,554	3,498	0.366(0.356∼0.376)	0.0%
DASS-21	3	6,766	1,449	0.226(0.186∼0.265)	91.9%
DSRSC	2	1,432	163	0.114(0.097∼0.130)	0.0%
**Grade**					
Included P	6	12,044	2,330	0.165(0.124∼0.206)	96.9%
Not included P	7	29,685	11,908	0.391(0.334∼0.447)	98.9%

*P, primary school students.*

Six studies included primary school students in the sample group, the pooled prevalence was estimated as 16.5% (95% CI, 12.4–20.6%; *I*^2^ = 96.9%). Seven studies did not include primary school students in the sample group, the pooled prevalence was estimated as 39.1% (95% CI, 33.4–44.7%; *I*^2^ = 98.9%).

### Reporting Biases

**FIGURE 3:**
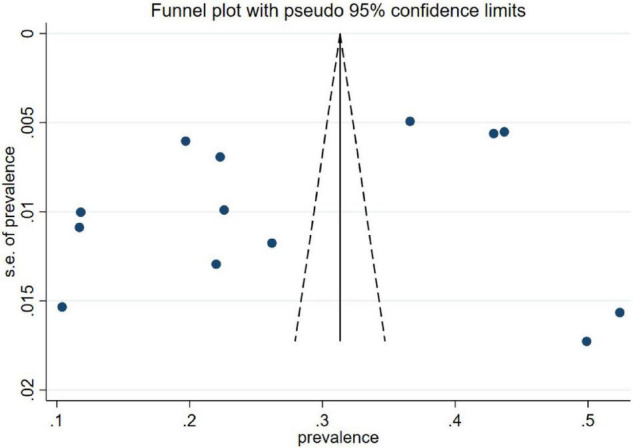
Funnel plot of depressive symptoms.

No obvious bias was found on visual inspection of the funnel plot (Figure 3). Egger’s and Begg’s quantitative analyses showed that there was no publication bias in the data set (*p* = 0.704 > 0.05).

### Sensitivity Analysis

**FIGURE 4:**
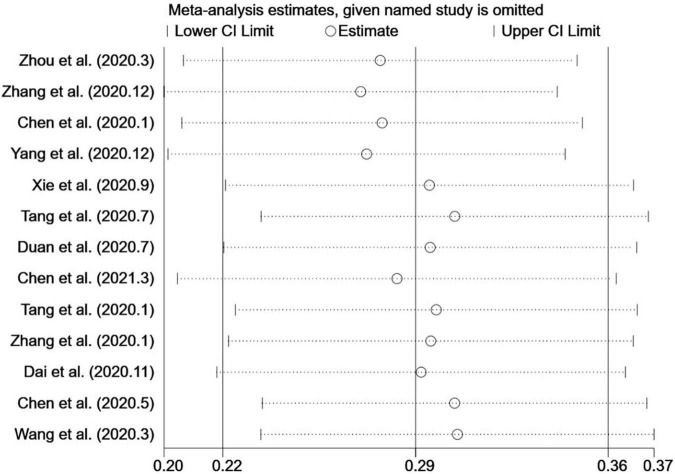
Sensitivity analysis chart.

Sensitivity analyses performed by consecutively omitting each study in each group showed that no single study significantly affected the initial results (Figure 4).

## Discussion

### Prevalence of Depressive Symptoms Among Children and Adolescents

This systematic review and meta-analysis of 13 original studies involving 41,729 participants showed that 10.4–52.4% of children and adolescents were screened positive for depression during the COVID-19 epidemic, and the pooled prevalence was 28.6%. This prevalence was almost the same as that of the general Chinese population during the COVID-19 outbreak, which was 28% (30) (12 original studies involving 27,475 individuals were included). In a meta-analysis of 29 studies that include 80,879 children and adolescents worldwide, the pooled prevalence of clinical depression was estimated as 25.2% (31). This result is similar to our study.

There are two possible reasons for the relative high prevalence of depressive symptoms among children and adolescents during the pandemic: First, the COVID-19 spreads *via* human-to-human transmission, children, and adolescents might worry about infections for themselves and their family members, especially when they had some common symptoms (such as a cough, runny nose, and headache) (32). Second, during the pandemic, home confinement also caused many adversely psychological impacts on children and adolescents, such as depression, anxiety and stress-related disorders (33). The online classes and e-learning could not provide the same environment as the live school (34) and social contact, security, and self-efficacy of students (35, 36). These might all contribute to the occurrence of depression in children and adolescents. While the experience of depression in adolescence is associated with a myriad of adult psychosocial outcomes, it may lead to the propagation of difficulties across the lifespan (37). We need to pay more attention to the mental health of children and adolescents during the pandemic.

In addition, before COVID-19, the prevalence of depression in children and adolescents worldwide was 12.9% (38), while in mainland China it was 22.2% (4) (62 original studies involving 232,586 children and adolescents). This suggests that the global prevalence of depression among children and adolescents during the COVID-19 pandemic has doubled when compared to pre-pandemic data (31), with only a slight increase in mainland China. The possible reasons for the slight increase in China are as follows: first, the pre-pandemic situation may contribute to this result. There was a high prevalence of depression in Chinese children and adolescents before COVID-19. As is known to all, the main purposes of education in Chinese schools are to obtain higher scores in the National College Entrance Examination with repeated exercises and examinations. School education stress is a serious social problem in China, many tests, exams, strict rules, and regulations in school, all could be stressors of depression (39–41). Second, the post-pandemic situation may deteriorate continually. A meta-analysis showed that the prevalence of depression symptoms among the general population was still increasing after the peak of COVID-19 in China (42). The possible reason is that the occurrence of depression requires a certain period of emotional accumulation and the occurrence of post-disaster depression has a certain delayed effect (43). While most studies included in this review were conducted in 2–3 months after COVID-19, the investigation time range was relatively narrow. The prevalence of depression may continue to increase during the continuing pandemic. As COVID-19 has continued for more than 2 years, the actual prevalence may be much higher than our result.

### Prevalence of Depressive Symptoms in Different Grade Groups

In subgroup analysis, we found that the pooled prevalence of depression in the 6 studies that include primary school students was 16.5%, and the pooled prevalence of depression in 7 studies that exclude primary school students was 39.1%. The result is consistent with previous studies. For instance, a previous meta-analysis demonstrated that the prevalence of depression among primary school students in China was 17.2% (27 studies that involve 42,374 subjects were included) (44), and the prevalence of depression among secondary school students was 24.3% (45) (51 original studies that involve 144,060 adolescents were included). We also found that in grade7 of secondary school, the prevalence was 24.5%, while in grade 10, the prevalence was increased to 40.1%.

The difference in the prevalence of depression between different grade groups may be related to biological changes, such as hormone changes during puberty (46). A meta-analysis indicated that depressive symptoms are dynamic during puberty. Depressive symptoms tend to increase in early adolescence, reach their peak during mid-adolescence, and then begin to decrease in the late adolescence (during the transition to adulthood) (47). Additionally, in primary and secondary schools, the academic difficulty is different. Adolescents in higher grades may experience more adjustment difficulties, such as academic problems and social deficits, and are therefore more likely to have depressive symptoms. Senior students (especially these facing university entrance exams) suffered more from abruptly coronavirus-related new challenges, i.e., uncertainty of the exam time and the date of returning school.

### Screening Scales for Evaluating Depression

In this study, we also found that a total of five different screening self-rating scales were used in cross-sectional studies that investigate the prevalence of depression among children and adolescents in China during the COVID-19 pandemic. Different depression screening scales have different clinical validity on the prevalence of depression (48).

Some previous studies have explored the application of CDI in children and adolescents in mainland China, showing that the internal consistency coefficient of CDI was between 0.82 and 0.88, and the test-retest reliability was between 0.75 and 0.89, which means it has acceptable reliability and validity (49). The sensitivity of the DSRSC for diagnosing depression among children and adolescents in China was 86%, and the specificity was 82% for the assessment (50). The DASS-21 has previously been used to assess children and adolescents who were aged 11–19 in China (51) and among these populations after the Sichuan earthquake (52). Previous studies also found that the CESD showed high reliability and stability in screening adolescents with depression (53). A large sample of Chinese children and adolescents completed the PHQ-9 in a cross-sectional survey (*N* = 10,933), the results indicated that the PHQ-9 is a reliable and valid scale and can be used in Chinese children and adolescents (54). The reliability and validity of the PHQ-9 scale in Chinese children and adolescents also have been tested, and it was found that the optimal cutoff of the PHQ-9 scale was 10 points, with a sensitivity of 93.33% and a specificity of 96.83% based on a receiver operator characteristic (ROC) curves (55).

In our analysis, the PHQ-9 was applied the most (4 studies that involved 17,707 participants), the pooled prevalence of depression assessed by the PHQ-9 scale was 46.8%, significantly higher than other scales (11.4% with DSRSC, 18.9% with CDI, 22.6% with DASS-21, and 36.6% with CESD). The PHQ-9 was applied by Zhou et al. (20), Zhang et al. (27), Chen et al. (29), and Yang et al. (22) in our study. They all adopted 5 points as the cutoff point for depression. This may be the reason for its higher detection rate of depression in the assessment of Chinese children and adolescents. To sum up, our results suggest that PHQ-9 may be a widely used self-assessing depression scale for children and adolescents in China, and 10 points may be an appropriate cutoff.

## Limitations

First, this study is considered to have heterogeneity. Since clinical and methodological diversities always occur in a meta-analysis, statistical heterogeneity is relatively inevitable (17). We have conducted subgroup analyses and performed a random-effects meta-analysis to avoid the effect of heterogeneity, as Cochrane recommends (56). During the COVID-19 pandemic, the severity of the epidemic and control measures varied among provinces in China, thus the prevalence of depression might vary in different provinces (57). Unfortunately, however, in our analysis, most of the studies did not provide accurate information about provinces, so we could not perform the analysis by province. We will continue to pay attention to this topic, there may be enough information to conduct the analysis by the province in the future. Second, due to the heterogeneity of screening scales, the evidence to support this estimated value of the depression prevalence is not strong enough. We will continue to update the data. If the data are sufficient, we will carry out a meta-analysis based on one scale, such as PHQ-9. The pooled prevalence rate will more truly reflect the situation.

## Conclusion and Future Directions

In summary, our findings show that depressive symptoms were frequently experienced by Chinese children and adolescents during the COVID-19 epidemic. Close attention should be paid to the mental status of children and adolescents, especially to the secondary school students. The strengthened psychoeducation, enhanced policy efforts, and effective medical services are needed. In the future, epidemiological studies on Chinese children and adolescents should focus on selecting scales that have good reliability and validity in this population and adapt appropriate cutoff points. In addition, it is worth to consider that performing a two-stage prevalence estimation (58) after the COVID-19 epidemic is fully controlled and ends.

## Data Availability Statement

The original contributions presented in the study are included in the article/supplementary material, further inquiries can be directed to the corresponding author/s.

## Author Contributions

JC and KY designed the study, conducted the statistical analysis, and wrote the manuscript. YC and JC conducted the double screening, coding, and assessed the quality of the reviewed studies. KY provided consultations for data analysis. MQ and NW critically reviewed and revised the manuscript. All authors provided critiques and approved the final manuscript.

## Conflict of Interest

The authors declare that the research was conducted in the absence of any commercial or financial relationships that could be construed as a potential conflict of interest.

## Publisher’s Note

All claims expressed in this article are solely those of the authors and do not necessarily represent those of their affiliated organizations, or those of the publisher, the editors and the reviewers. Any product that may be evaluated in this article, or claim that may be made by its manufacturer, is not guaranteed or endorsed by the publisher.
